# Tranexamic Acid for Blood Loss after Transforaminal Posterior Lumbar Interbody Fusion Surgery: A Double-Blind, Placebo-Controlled, Randomized Study

**DOI:** 10.1155/2020/8516504

**Published:** 2020-08-14

**Authors:** Bin He, Yuanqiang Li, Shuai Xu, Yunsheng Ou, Jinqiu Zhao

**Affiliations:** ^1^Department of Orthopedics, The First Affiliated Hospital of Chongqing Medical University, Chongqing, China 400016; ^2^Department of Infectious Diseases, The First Affiliated Hospital of Chongqing Medical University, Chongqing, China 400016

## Abstract

**Background:**

Transforaminal lumbar interbody fusion (TLIF) may result in significant blood loss and an increase in blood transfusion. Though tranexamic acid (TXA) is widely studied for the hemostasis of arthroplasty, there is little information on the use of TXA for TLIF surgery.

**Methods:**

This prospective randomized, double-blind, placebo-controlled trial was conducted to study the influence of TXA (intravenous bolus of 10 mg/kg 15 minutes before skin incision followed by intravenous infusion of 6-8 mg/kg/h up to a total dose of 15 mg/kg during the surgery) on the blood loss and Enhanced Recovery After Surgery (ERAS) after TLIF surgery. 40 patients were randomized into two groups: TXA group (tranexamic acid) and control group (placebo). Baseline characteristics were comparable between the TXA group and the control group before the surgery. Outcomes assessed included blood loss, total postoperative drainage, time for drainage removal, time to ambulation, hospital stay after surgery, postoperative hemoglobin (Hb) one day after surgery, and adverse events.

**Results:**

Compared to patients in the control group after TLIF surgery, patients in the TXA group have significantly reduced intraoperative hemorrhage and time to ambulation after surgery but show similar hospital stay, postoperative drainage, time for drainage removal, postoperative Hb one day after surgery, and adverse events.

**Conclusions:**

TXA shows important ability in controlling blood loss and promoting the ERAS after TLIF surgery.

## 1. Introduction

With the increase in aging population, degenerative lumbar diseases (e.g., lumbar disc herniation and spondylolisthesis) have high morbidity [1–3]. Transforaminal lumbar interbody fusion (TLIF) has been widely accepted to treat these diseases and is associated with less trauma and blood loss compared to posterior lumbar interbody fusion [4–6]. However, extensive paravertebral muscle stripping and retraction is still needed to obtain an adequate surgical field during TLIF [7–10].

Because the anatomical structures of the spine have spongy vertebrae with rich blood supply and fragile venous plexus, substantial blood loss frequently occurs during the TLIF and increases postoperative morbidity and prolongs clinical recovery [11–13]. Tranexamic acid (TXA), a synthetic lysine analogue of *trans*-4-aminomethyl-cyclohexane-1-carboxylic acid, has been studied to reduce intraoperative blood loss and the need for blood transfusion [14].

Some studies confirmed the efficacy of TXA for controlling blood loss in total knee arthroplasty and total hip arthroplasty [15, 16]. Only two studies reported the combination use of TXA by preoperative bolus loading and continuous infusion maintenance [17, 18]. To our knowledge, this prospective randomized, double-blind, placebo-controlled trial is the first study to focus on the safety and effect of TXA combination use (intravenous bolus of 10 mg/kg 15 minutes before skin incision followed by intravenous infusion of 6-8 mg/kg/h up to a total dose of 15 mg/kg during the surgery) on blood loss reduction in TLIF. In particular, ERAS outcomes (e.g., time to ambulation and hospital stay) are evaluated between TXA and placebo.

## 2. Material and Methods

### 2.1. Study Design

After obtaining the approval of the Committee of Medical Ethics and the institutional review boards of our institutions, we designed and conducted a randomized, prospective, double-blind, and placebo-controlled trial at our institute from July 2018 to February 2019 following the Consolidated Standards of Reporting Trials (CONSORT) guidelines. Patients or the public were not involved in the design, conduct, reporting, or dissemination plans of our research.

40 patients were randomly allocated into the TXA group (tranexamic acid) or control group (placebo) using computer-based random number generation technique. The TXA group (tranexamic acid) received intravenous bolus of TXA 10 mg/kg 15 minutes before skin incision followed by intravenous infusion of 6-8 mg/kg/h up to a total dose of 15 mg/kg during the surgery. The control group received the equal volume of 0.9% normal saline as placebo. We obtained the informed written consent from each patient.

BH was responsible for the randomization of the project and the concealing of TXA by the unified packaging, YQL and SX implemented the intervention of each patient, and JQZ and YSO observed patients and collected the outcome data. The patients, researchers (YQL, SX, JQZ, and YSO), surgeon, and anesthesiologist were blinded to the patients' treatment. At the end of data collection, BH analyzed the data by groups.

The eligibility criteria were as follows: (1) age between 30 and 80 years old; (2) lumbar disc herniation, stenosis, or spondylolisthesis with unilateral radiculopathy; and (3) one-level or two-level TLIF surgery. The exclusion criteria included lumbar fracture, previous spinal surgery, deformities requiring the correction, coagulation disorder, anticoagulants, or antiplatelet medications. All operations were done by a single senior surgeon. General anesthesia was applied for all patients.

### 2.2. Surgical Technique

Lumbar interbody fusion surgery was conducted through posterior midline skin incision. Subperiosteal exposure of respective levels was done in the decompression side in order to expose the facet joints. The facet joints in the contralateral side were exposed via separating muscular space between the longissimus and multifidus muscles to expose the facet joint of corresponding levels. Pedicle screws were placed with freehand technique, and connecting rods with an adequate size were installed.

After lamina resection, removal of the corresponding disc, and decompression of nerve root transforaminally, the disc space was distracted to the appropriate height for the insertion of a cage. A cage filled with autogenous bone graft was obliquely inserted into the intervertebral disc space. The pedicle screws and final placement of the cage were confirmed by radiography. After achieving copious irrigation and hemostasis, drainage catheters were placed, and the wounds were closed layer by layer. An intermittent pneumatic compression device was used for the prophylaxis of deep vein thrombosis (DVT) after the surgery.

### 2.3. Outcome Measures

We recorded the baseline characteristics of each patient and included the age of patients, sex, body mass index (BMI), Hb, hematocrit (HCT), platelet (PLT), prothrombin time (PT), activated partial thromboplastin time (APTT), alanine transaminase (ALT), aspartate transaminase (AST), and surgical time.

Many important outcomes were compared between the two groups, and they involved intraoperative blood loss, total postoperative drainage, time for drainage removal, time to ambulation, hospital stay after surgery, and postoperative Hb one day after surgery. The drainage removal was conducted when the drainage fluid for 24 h was less than 30 ml. In addition, blood transfusion and adverse events were recorded. Time to ambulation indicated the time period from the end of surgery to the day when patients could walk, while hospital stay after surgery represented the time period from the end of surgery to patient discharge.

### 2.4. Sample Size

In determining the sample size of the study based on the preliminary experimental results of intraoperative blood loss (90 ± 21.1 ml in the TXA group versus 155 ± 79.8 ml in the control group), the power was equal to 80% and type I error rate was 0.05. The final sample sizes in each group were 17 or greater, and thus, this study was to recruit 20 patients in each group.

### 2.5. Statistical Analysis

All parametric data were presented as mean ± standard deviation (SD). Student's *t*-test for continuous variables or the chi-squared test for dichotomous variables was used to find the significance of study parameters between the TXA group and the control group. These two tests were used to compare the demographic parameters and perioperative and postoperative parameters between the two groups. *P* < 0.05 was thought to be statistically significant.

## 3. Results

40 patients were allocated in two groups. There were 15 cases with one-level lumbar interbody fusion and 5 cases with two-level lumbar interbody fusion in each group. Baseline demographic parameters such as age, sex, BMI, Hb, HCT, PLT, PT, APTT, ALT, AST, and surgical time were comparable between the two groups (*P* > 0.05, Table 1). The total dose of tranexamic acid was 1631.88 ± 311.87 mg in the TXA group.

The mean intraoperative blood loss of the TXA group was 91.50 ± 37.31 ml, which was significantly lower than 145 ± 108.7 ml in the control group (*P* = 0.04). In contrast, there was no statistical significance of postoperative drainage between the TXA group and the control group (147.7 ± 70.47 ml versus 157.35 ± 68.3 ml, *P* = 0.4). No significant difference was observed between the two groups in terms of time for drainage removal (3.25 ± 0.55 days versus 3.00 ± 0.73 days, *P* = 0.23, Table 2).

**Figure 1:**
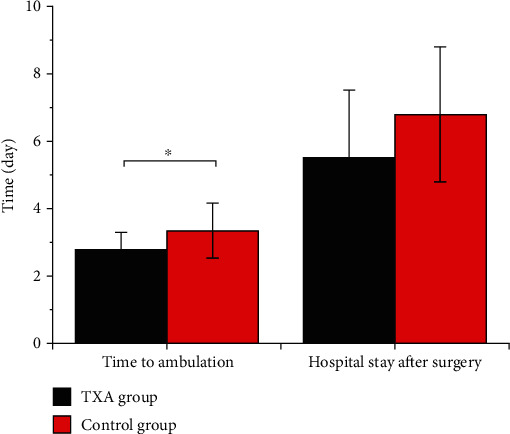
Comparison of time to ambulation and hospital stay after surgery (day) between the two groups. ∗ represents *P* < 0.05.

**Figure 2:**
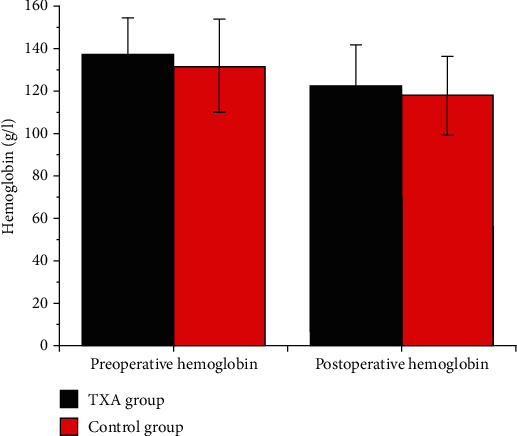
Comparison of preoperative and postoperative hemoglobin (Hb, g/l) one day after surgery between the two groups.

Furthermore, patients in the TXA group required less time to ambulation (2.8 ± 0.52 days versus 3.35 ± 0.81 days, *P* = 0.049) compared to patients in the control group but showed comparable hospital stay after surgery (5.5 ± 2.0 days versus 6.8 ± 1.99 days, *P* = 0.54, Figure 1). No patients in the two groups needed blood transfusion (Table 2). Additionally, the postoperative Hb one day after surgery in the TXA group was similar to that in the control group (122.6 ± 19.3 g/l versus 117.75 ± 19.03 g/l, *P* = 0.43, Figure 2).

Adverse events were found in 9 patients (5 cases in the TXA group, 4 cases in the control group). Superficial wound infection was observed in one patient of each group, and these two patients obtained wound healing after debridement and suturing under local anesthesia. Two patients suffered from hypoproteinemia (one case in each group). In addition, liver dysfunction was found in 3 patients of the TXA group and 2 patients in the control group. These patients all achieved recovery after drug therapy. No DVT was found. The total complications were similar in the two groups (*P* = 0.71, Table 2).

## 4. Discussion

The procedures of lumbar spinal fusion surgery include the decompression, instrumentation, correction, and fusion. Intraoperative blood loss during lumbar fusion surgery is estimated to be over 800 ml (range 100~3100 ml) for noninstrumented fusion and 1517 ml (range 360~7000 ml) for instrumented fusion [19]. Blood transfusion is occasionally required to treat symptomatic anemia and promote postoperative rehabilitation [20]. Adequate hemostasis can reduce the risk of epidural hematoma formation, which may cause neural compression and neurological deficits [21].

Many methods have been developed to control bleeding during spinal surgery and mainly include minimal invasion procedures, patient positioning, deliberate hypotension, intra-abdominal pressure control, infiltration of paraspinal tissues using vasoconstrictors, and pharmacological agents to enhance coagulation [10, 22–25]. TXA is known as an antifibrinolytic agent and acts through blocking the interaction of plasminogen and plasmin by competing with the lysine residues on the surface of fibrin to inhibit the fibrinolysis, resulting in clot stabilization [26–29]. Our study suggests that preoperative and intraoperative intravenous infusion of TXA can substantially reduce intraoperative blood loss and time to ambulation but shows no obvious impact on hospital stay after surgery, postoperative drainage, time for drainage removal, or postoperative Hb.

Only two RCTs reported the impact of preoperative and intraoperative intravenous infusion of TXA on blood loss for spinal fusion surgery [17, 18]. Intravenous tranexamic acid might have the better ability to reduce blood loss than its local infiltration [26]. Low dose (5 mg/kg of bolus loading dose and 1 mg/kg of continuous infusion until 5 h after surgery) and high dose (10 mg/kg of bolus loading dose and 2 mg/kg of continuous infusion until 5 h after surgery) of TXA were applied for single-level posterior lumbar interbody fusion, and 24 cases were included in each group. The results found that TXA resulted in the significant decrease in intraoperative blood loss (385 ± 139 ml versus 542 ± 333 ml in the control group, *P* = 0.03), but there was no statistical difference between the low dose of TXA and the control group (508 ± 269 ml versus 542 ± 333 ml, *P* = 0.74), indicating that high dose of TXA was effective to reduce blood loss for posterior lumbar interbody fusion [18]. Considering the outcomes including Hb and HCT, high dose of TXA and control intervention resulted in similar change of Hb (1.3 ± 0.6 versus 1.7 ± 0.2 g/dl, respectively, *P* = 0.75) and HCT (2.3 ± 1.6 versus 5.8 ± 2.3%, respectively, *P* = 0.15) [18], which was also confirmed by another study involving 50 patients in the TXA group and 46 patients in the control group for posterior lumbar surgery [17].

This RCT investigates the influence of higher dose (10 mg/kg 15 minutes before skin incision followed by intravenous infusion of 6-8 mg/kg/h up to a total dose of 15 mg/kg during the surgery) of TXA for TLIF than the study conducted by Kim et al. [18]. Theoretically, the better efficacy to reduce blood loss should be observed in our results, but the mean intraoperative blood loss of the two groups was 91.50 ± 37.31 ml for TXA and 145 ± 108.7 ml for placebo, respectively. The *P* value between groups was only equal to 0.04. These may be caused by the small patient sample and the difference in blood loss due to various levels of surgical trauma. Additionally, no statistical difference in postoperative Hb remained between the two groups in our results, which was consistent with previous two studies [17, 18], possibly because blood loss of this surgery was not great. Patients can immediately and partially recover from the blood loss, which could not be obviously presented in postoperative Hb.

Our study revealed no incidence of DVT. The total adverse events were similar between the TXA group and the control group. Our results confirmed the efficacy and safety of this TXA for spinal fusion. In addition, time to ambulation after surgery was remarkably reduced by TXA administration, which promotes ERAS. However, the results of intraoperative blood loss and postoperative Hb were inconsistent, possibly because the amount of blood loss in TLIF was not very high. Patients had sufficient recovery ability after blood loss, and thus, no significant difference of postoperative Hb was observed between the two groups. The studies regarding complex spinal surgeries (e.g., spinal tuberculosis or deformity correction) with higher amount of blood loss should be conducted to explore the impact of TXA.

## 5. Limitations

Our study still has several limitations. Firstly, the blood loss in TLIF is relatively low, and future studies should focus on the impact of TXA on complex spinal surgeries with high amount of blood loss. Secondly, the patients in each group have no preoperative or postoperative anemia, and thus, the potential of TXA in reducing blood transfusion cannot be investigated in this study. Thirdly, no DVT is found in the two groups, and we need to explore this dose of TXA on thromboembolism in patients with high risk.

## 6. Conclusion

Intravenous TXA provides additional benefits to blood loss reduction and ERAS in patients with TLIF.

## Figures and Tables

**Table 1:** Demographic data and clinical characteristics.

Variable	TXA group	Control group	*P* value
Number	20	20	
Age (year)	57.95 ± 12.44	57.9 ± 11.76	0.99
Male/female	8/12	11/9	0.34
BMI (kg/m^2^)	25.02 ± 5.19	24.75 ± 4.42	0.86
Hb (g/l)	137.65 ± 16.88	131.95 ± 22.45	0.40
HCT (%)	40.86 ± 4.41	39.24 ± 5.88	0.33
PLT (10^9^/l)	183.85 ± 54.68	174.35 ± 53.87	0.58
PT (s)	12.92 ± 2.2	12.95 ± 0.70	0.95
APTT (s)	34.26 ± 4.28	36.45 ± 3.31	0.08
ALT (U/l)	30.65 ± 38.31	24.45 ± 19.02	0.52
AST (U/l)	22.55 ± 14.02	24.5 ± 15.62	0.68
Surgical time (min)	159.20 ± 29.91	143.65 ± 36.83	0.15

TXA: tranexamic acid; BMI: body mass index; Hb: hemoglobin; HCT: hematocrit; PLT: platelet; PT: prothrombin time; APTT: activated partial thromboplastin time; ALT: alanine transaminase; AST: aspartate transaminase.

**Table 2:** Comparison of clinical outcomes between the TXA group and the control group.

Variable	TXA group	Control group	*P* value
Intraoperative blood loss (ml)	91.50 ± 37.31	145 ± 108.7	0.04
Postoperative drainage (ml)	147.7 ± 70.47	157.35 ± 68.3	0.4
Time for drainage removal (day)	3.25 ± 0.55	3.00 ± 0.73	0.23
Blood transfusion	0	0	—
Adverse events	5	4	0.71

TXA: tranexamic acid.

## Data Availability

The datasets generated during and/or analyzed during the current study are available in the ResMan Research Manager repository (http://www.medresman.org.cn/uc/sindex.aspx).
